# Novel Textile Scaffolds Generated by Flock Technology for Tissue Engineering of Bone and Cartilage

**DOI:** 10.3390/ma5030540

**Published:** 2012-03-22

**Authors:** Anja Walther, Birgit Hoyer, Armin Springer, Birgit Mrozik, Thomas Hanke, Chokri Cherif, Wolfgang Pompe, Michael Gelinsky

**Affiliations:** 1 Max Bergmann Center of Biomaterials, Institute for Materials Science, Technische Universität Dresden, Budapester Str. 27, Dresden 01069, Germany; E-Mails: walther.anja@gmx.net (A.W.); thomas.hanke@tu-dresden.de (T.H.); wolfgang.pompe@nano.tu-dresden.de (W.P.); 2 Centre for Translational Bone, Joint and Soft Tissue Research, Medical Faculty of Technische Universität Dresden, University Hospital Carl Gustav Carus, Fetscherstr. 74, Dresden 01307, Germany; E-Mails: birgit.hoyer@tu-dresden.de (B.H.); armin.springer@tu-dresden.de (A.S.); 3 Ingenieurbüro, Voglerstr. 33, Dresden 01277, Germany; E-Mail: birgit@mrozik.de; 4 Institute of Textile Machinery and High Performance Material Technology, Technische Universität Dresden, Dresden 01062, Germany; E-Mail: chokri.cherif@tu-dresden.de

**Keywords:** tissue engineering, flock scaffold, flock technology, bone, cartilage

## Abstract

Textile scaffolds can be found in a variety of application areas in regenerative medicine and tissue engineering. In the present study we used electrostatic flocking—a well-known textile technology—to produce scaffolds for tissue engineering of bone. Flock scaffolds stand out due to their unique structure: parallel arranged fibers that are aligned perpendicularly to a substrate, resulting in mechanically stable structures with a high porosity. In compression tests we demonstrated good mechanical properties of such scaffolds and in cell culture experiments we showed that flock scaffolds allow attachment and proliferation of human mesenchymal stem cells and support their osteogenic differentiation. These matrices represent promising scaffolds for tissue engineering.

## 1. Introduction

The use of fibers as material for sutures in surgery is a very long-standing and also simple application of textiles in medicine. By the use of certain textile technologies it is also possible to generate complex structures and three dimensional designs.

The reason why textiles are used in regenerative medicine and tissue engineering is that they exhibit many advantages over other methods to fabricate scaffolds. One is the excellent surface-to-volume ratio of fibers and therefore also of the resulting textile structures. With their large surface, fibers offer a huge area for cell adhesion and are therefore effective for the cultivation of cells. By varying the fiber diameter or distance, as well as surface properties of the fibers, the characteristics of scaffolds can be changed very easily. Different textile technologies like electrospinning [1,2,3], weaving, knitting, *etc*. allow for the variation of the properties and the adaptation to the particular application [4]. Ramakrishna has reviewed the variety of textile technologies which may be utilized for scaffold production [5].

### 1.1. Flock Technology

Flock technology is a well-known and often applied textile technology. Originally developed for the improvement of visual appearance and haptics, flocking can nowadays also be found in technical applications.

**Figure 1:**
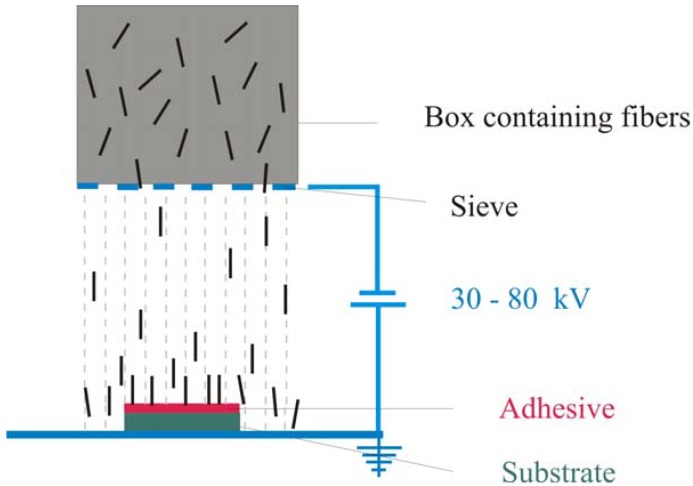
Schematic representation of the flocking process.

Electrostatic flocking means to apply short fibers on a substrate that is covered with adhesive in an electrical field so that the fibers are nearly vertical to the substrate [6]. In the electrostatic field the fibers are aligned and accelerated towards the adhesive coated substrate (Figure 1). Reaching the adhesive layer the fibers become stuck perpendicularly to the substrate and give the surface a velvet-like look.

Our aim was to use flock technology for the fabrication of a new type of scaffold for tissue engineering. The purpose of a tissue-engineered scaffold is to mimic the properties of the extracellular matrix (ECM) of the tissue that should be restored as well as possible and temporarily adopt its function. For that to happen a scaffold has to be mechanically stable, serve as matrix for cell adhesion, proliferation and differentiation, allow nutrient and oxygen support and provide enough space for newly synthesized matrix and blood capillary ingrowth. Most tissues in the body exhibit anisotropic properties so that anisotropic replacement materials are better suited than isotropic ones.

In previous experiments we have adapted flock technology from the textile industry for the fabrication of scaffolds for tissue engineering by successfully replacing the adhesive and the substrate with biocompatible and degradable materials [7]. We could also show that the scaffolds were stable under cell culture conditions.

The aim of the present study was to create scaffolds with different properties by varying both flocking fiber geometry and flocking time. The generated scaffolds were characterized with regard to their biomechanical properties. In cell culture experiments they were loaded with human mesenchymal stem cells to investigate the ability of cells to differentiate into osteoblasts in flock scaffolds.

## 2. Results and Discussion

To generate the flock scaffolds for this study a membrane made from mineralized collagen was used as substrate and a gelatine solution utilized as adhesive. Because of the lack of resorbable fibers, applicable for electrostatic flocking, the presented scaffolds consisted of flock fibers made of polyamide which are frequently used for flocking in industry. These fibers are biocompatible and were used in the experiments as model-fibers until suitable biodegradable fibers will be available. By varying flocking times—*i.e.*, the periods of time in which the fibers were shot at the substrate—scaffolds with different flock densities were achieved.

### 2.1. Microscopic Investigation of Different Scaffold Types

**Figure 2:**
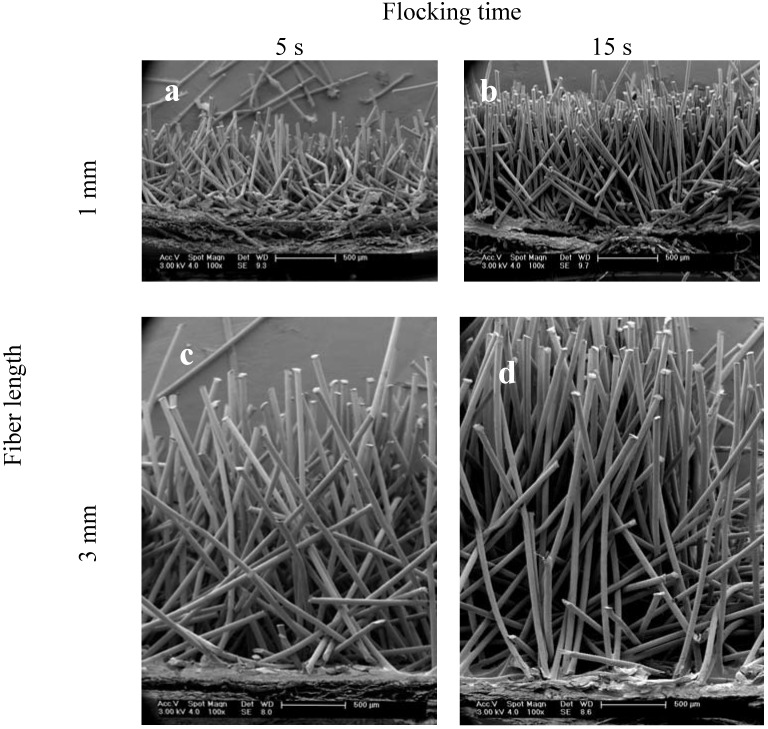
Scanning electron microscopy (SEM) images of scaffolds produced with different flocking times and fiber lengths. The figure shows representative images of longitudinal cuts of the four different scaffold types. (**a**) 1 mm fiber length, 5 s flocking time; (**b**) 1 mm fiber length, 15 s flocking time; (**c**) 3 mm fiber length, 5 s flocking time and (**d**) 3 mm fiber length, 15 s flocking time. Magnification: 100×.

For the fabrication of the scaffolds, two types of fibers (differing in length and diameter) and varying flocking times were used. By using different fiber lengths structures with variable heights could be produced. Varied flocking times resulted in distinct flock densities (and therewith fiber distances). Flocking times were varied between 5 and 15 s. Figure 2 shows typical longitudinal cuts through all of the four produced scaffold types. On the cutting edge, fibers were inclined because of the sectioning of the samples with a razor blade.

Microscopic investigations revealed that shorter flocking times of 5 s resulted in scaffolds with a less dense structure and less oriented fibers (Figure 2a,c) compared to scaffolds that were produced using 15 s flocking time (Figure 2b,d). For both short and long fibers the orientation of the fibers increased with longer flocking time. The different heights of the scaffolds according to the fiber length used can be seen in the images. The best alignment of fibers was found in the scaffolds with 1 mm fiber length and 15 s flocking time (Figure 2b).

The nearly parallel arrangement of the fibers results in scaffolds with an oriented pore structure. This might be favorable for engineering of articular cartilage because this tissue shows a column-like orientation of cells.

### 2.2. Flock Density

**Figure 3:**
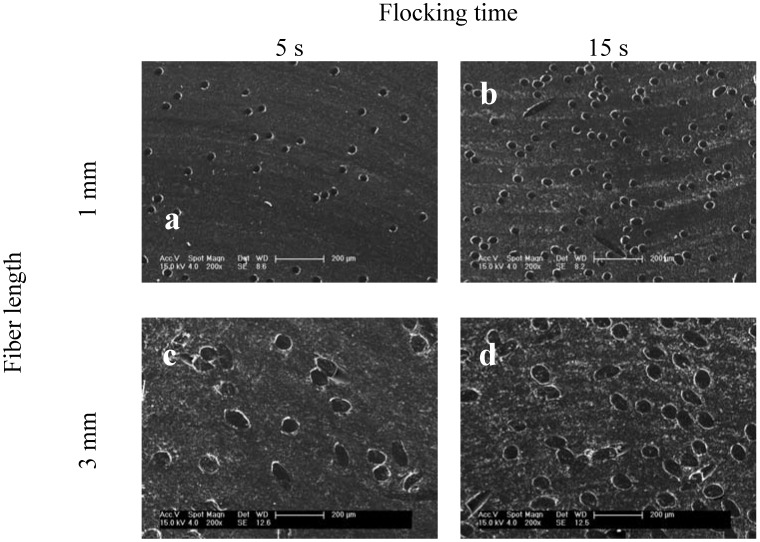
SEM images of four scaffold types showing different flock densities. Presented are cross-sections from samples that were embedded in epoxy resin and then trimmed. Micrographs represent typical results from scaffolds with (**a**) 1 mm fiber length, 5 s flocking time; (**b**) 1 mm fiber length, 15 s flocking time; (**c**) 3 mm fiber length, 5 s flocking time and (**d**) 3 mm fiber length, 15 s flocking time. SEM images at a magnification of 200×.

The flock density—fibers per unit area—is a parameter that allows for a statement to be made about the pores and the porosity of the scaffolds, which is important for cell ingrowth. The flocking density increased with flocking time and smaller fiber diameter. Cross-sections of the four scaffold types produced are shown in Figure 3.

The cross-sections of the scaffolds give information about the pores and the pore range of the samples. In case of flock scaffolds, a pore is defined as the space between the fibers. Figure 3 shows that flock scaffolds have large pores which are highly interconnected. The pore size could easily be adjusted by the flocking time. The fact that there are huge pores combined with oriented fibers (Figure 2) should facilitate cell infiltration and makes flock scaffolds superior to scaffolds made by other textile technologies such as electrospinning.

Porosity is another characteristic of scaffolds for tissue engineering applications. We estimated the porosity of the produced scaffolds using the following equation:

(1) $$Porosity=\frac{V_{s}-V_{f}}{V_{s}}100\%$$

Porosity was calculated taking the volume of the scaffold ($V_{s}$) which is given by the fiber length and the diameter of the substrate, and the volume of all fibers in the scaffold ($V_{f}$) which can be estimated by the volume of a single fiber multiplied by the number of fibers per area that is given by the flock density. For the four investigated scaffold types the following porosities were evaluated (Table 1):

**Table 1:** Flocking density and porosity of the four different scaffold types.

Fiber length [mm]	Flocking time [s]	Flock density [fibers/mm^2^]	Calculated porosity of the scaffold [%]
1	5	72 ± 11.2	94.9 ± 0.8
1	15	104.5 ± 10.7	92.6 ± 0.8
3	5	11.4 ± 1.8	97.6 ± 0.4
3	15	21 ± 3.3	95.9 ± 0.65

For all of the introduced flock scaffolds the values for the calculated porosities are over 90 %. This illustrates that these scaffolds are highly porous structures. This could also be deducted by looking at the microscopic images of the embedded scaffolds in Figure 3. As expected, the prolonged flocking time of 15 s resulted in lower porosities than the short flocking time of 5 s. The highest porosity was found for scaffolds with long fibers (3 mm) and a short flocking time of 5 s.

Scaffolds with high porosities are considered particularly suitable for tissue engineering applications. Pores should be big enough and highly interconnected to provide enough space for cells to attach and to facilitate migration of cells into the scaffold. They have to ensure that cells are supplied with nutrients and oxygen but have also to enable the export of metabolic waste products. Looking on the porosity in conjunction with the size of the pores flock scaffolds are superior to nanofibrous mats made by electrospinning, commonly investigated nowadays for utilization in tissue engineering. Nanofibrous mats also show high porosity and resemble the extracellular matrix of tissues in morphology but they are mostly flat sheets that only possess pores in the lower micrometer range which can hinder deep cellular infiltration [8].

Apart from high porosities mechanical strength is also considered relevant for tissue engineering scaffolds to provide at least an initial stability of the construct after implantation.

### 2.3. Biomechanical Characterization of the Scaffolds

#### 2.3.1. Theoretical Model—Calculation of Mechanical Strength

**Figure 4:**
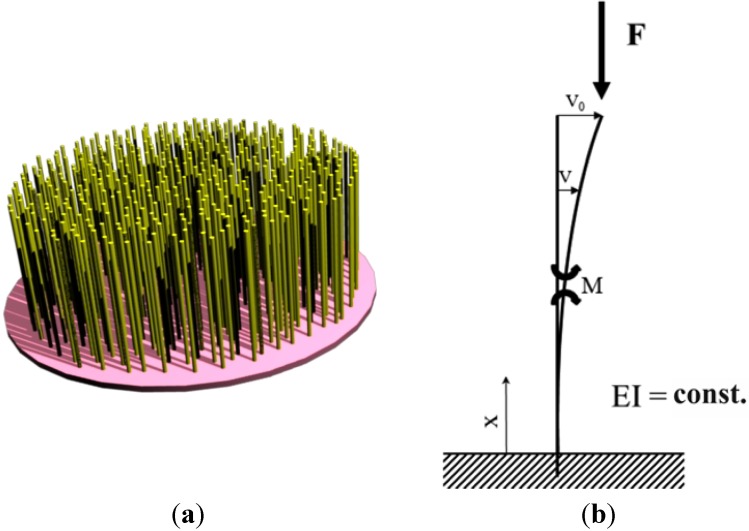
Model of the flock scaffold. (**a**) Theoretically optimal model of the flock scaffold; (**b**) Balance of forces on the fiber (according to [9]).

Prior to compression tests we did a stability analysis. For this purpose, we used the model of the scaffold shown in Figure 4 and calculated the expected compression strength for each of the four scaffold types described above.

Under the influence of compression forces, as shown in Figure 4, the fiber is exposed to a bending load which can be set up as follows:

(2) $$M=-F(v_{0}-v)$$

M is the bending moment, F is the compression force, v is the displacement and $v_{0}$ is the maximal displacement on top of the fiber.

The transposed differential equation of the bending line is:

(3) M=−EIv″

with E as module of elasticity and I as geometrical moment of inertia.

From Equations (2) and (3) it follows that

(4) $$v\prime \prime +\frac{F}{EI}v=\frac{F}{EI}v_{0}$$

For further calculation a simplification is made by introducing κ:

(5) $$\kappa^{2}=\frac{F}{EI}$$

From Equation (4) it then follows that

(6) $$v\prime \prime +\kappa^{2}v=\kappa^{2}v_{0}$$

To solve Equation (7), the following can be set up:

(7) $$v(x)=A\cos(\kappa x)+B\sin(\kappa x)+v_{0}$$

The boundary conditions (with *l* as length of the fiber)

(8) $$v(x=0)=0,v\prime (x=0)=0,v(x=l)=v_{0}$$

Led to the following system of equations:

(9) $$\begin{array}{l} A+v_{0}=0\\ B\kappa =0\\ A\cos\kappa l+B\sin\kappa l=0 \end{array}$$

Resulting in a matrix

(10) $$\begin{array}{ccc} 1 & 0 & 1\\ 0 & \kappa & 0\\ \cos\kappa l & \sin\kappa l & 0 \end{array}=-\kappa l\cos\kappa l=0$$

Which leads to:

(11) $$\kappa l=\frac{\pi }{2}$$

Hence the equation for calculating the critical compression force $F_{k}$ is:

(12) $$F_{k}=\frac{\pi^{2}EI}{4{l_{k}}^{2}}$$

Where *l_k_* is the length of the fibers that is not covered with adhesive.

From this, it follows that the compression strength of a single fiber $\sigma_{sf}$ is:

(13) $$\sigma_{sf}=\frac{F_{k}}{A_{f}}$$

With the microscopically evaluated flock density (number of fibers per area with *n_f_* as number of fibers, *A_f_* as area of fibers, and *A_ges_* as overall-area) the critical compression strength of a scaffold $\sigma_{k}$ could be calculated as follows:

(14) $$\sigma_{k}=\frac{n_{f}A_{f}}{A_{ges}}\sigma_{sf}$$

**Figure 5:**
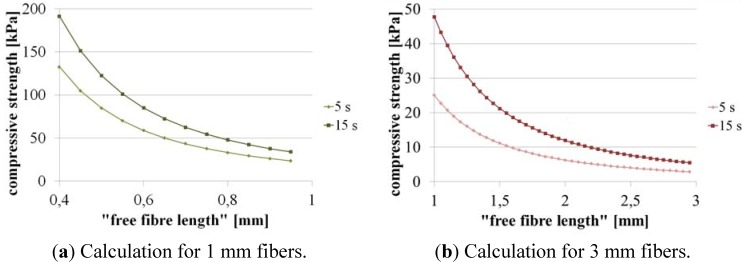
Calculated compression strength for the different flock scaffolds as a function of the “free fiber length”. (**a**) 1 mm fibers; (**b**) 3 mm fibers.

For the calculation of the critical compression strength of the scaffolds it is necessary to know how much of the fiber is covered with adhesive and how much “free fiber length” remains (the part of the fiber which is not integrated in the adhesive layer). Due to the fact that it is not possible to exactly measure the “free fiber length” we calculated the critical compression strength for different values (Figure 5).

The graphs show that the critical compression strength increases with shorter fibers and higher flock density. From microscopic images we estimated for the scaffolds with 1 mm fiber length a free fiber length of 0.8–0.95 mm so that the expected values for compression strength should be about 30–50 kPa whereas for the scaffolds with 3 mm fiber length (estimated free fiber length of 2.3–2.8 mm) it should be about 4–10 kPa. The results show that scaffolds with shorter fibers (1 mm; Figure 5a) should be more pressure-resistant than scaffolds with 3 mm fibers.

#### 2.3.2. Experimental Results of Compression Testing

In compression tests we evaluated the mechanical strength of flock scaffolds in wet state under unidirectional compressive loading. We compared their compression strength with the above calculated data.

The mechanical investigations showed that the different scaffold types exhibit different compression strengths as a function of fiber length and flock density. The measured values matched the values which were earlier calculated in the stability analysis (Table 2). The scaffolds made of 1 mm fibers and high flock density (15 s flocking time) were found to be the most pressure-resistant.

**Table 2:** Comparison of calculated and measured values for compression strength of flock scaffolds.

Scaffold parameters (fiber length—flocking time)	“Free fiber length” [mm]	Calculated compression strength [kPa]	Measured compression strength [kPa]
1 mm—5 s	0.8–0.95	30–40	34 ± 7
1 mm—15 s	0.8–0.95	40–50	36 ± 6
3 mm—5 s	2.3–2.8	4–6	10 ± 3
3 mm—15 s	2.3–2.8	6–10	25 ± 7

**Figure 6:**
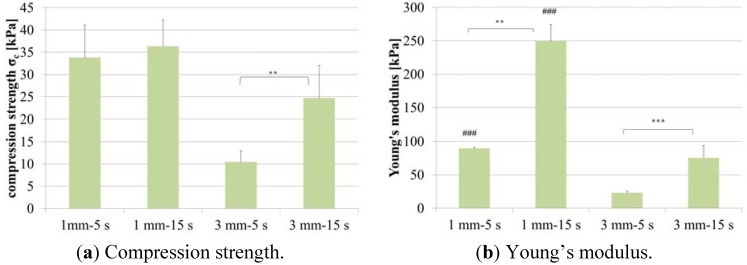
Mechanical characterization of four different flock scaffold types. (**a**) Compression strength; (**b**) Young’s modulus. Graph shows the mean (n = 5) and standard deviation of the mean. *** *p* < 0.001; ** *p* < 0.01; ### *p* < 0.001 (1 mm <-> 3 mm).

The differences between the scaffold types become better visible in Young’s modulus diagram (Figure 6b). Scaffolds with 1 mm fibers that were flocked for 15 s have a Young’s modulus of about 250 kPa which is the highest value for the investigated types of novel materials.

The nearly parallel arrangement of the fibers that are aligned perpendicular to the substrate results in mechanically stable structures. The fibers undergo Euler buckling and enable the fabrication of anisotropic structures with high compression strength despite their high porosity.

Janjanin *et al.* reported about electrospun scaffolds seeded with cells. In compression tests they investigated the mechanical properties of these structures and could show that Young’s modulus of the scaffolds rises up to about 17 kPa after 42 days of culture [10], which still is significantly lower than Young’s modulus of flock scaffolds without cells.

Concerning biomechanical properties of cell seeded flock scaffolds co-workers of us investigated the hardness of scaffolds seeded with human mesenchymal stem cells over a period of 42 days. They showed that the hardness of the constructs rises significantly during culture due to the deposition of new matrix by the cells [11]. Scaffolds for tissue engineering applications should be able to protect cells from early critical mechanical forces until enough matrix is synthesized by the cells to further stabilise the structure.

### 2.4. Microscopic Evaluation of Cell Seeded Flock Scaffolds

Human mesenchymal stem cells were seeded onto the scaffolds. Adherence and proliferation were microscopically investigated using scanning electron microscopy (SEM) as well as confocal laser scanning microscopy (cLSM).

**Figure 7:**
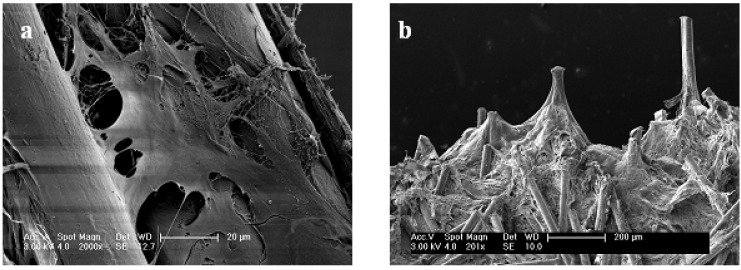
Scanning electron microscopy images of cell seeded samples (1 mm fiber length and 15 sec flocking time). (**a**) Single cell that stretches between two fibers; (**b**) Scaffold after 28 days of cultivation. All spaces between the fibers are filled with cells and newly deposited ECM.

Cells easily attached to the flock scaffolds and spread on as well as between the fibers (Figure 7a). Due to the highly interconnected pores with adequate pore size cells can easily distribute over the scaffolds. Otherwise the distance between the fibers is also small enough for the cells to spread and build bridges between the fibers. SEM investigations after 28 days of cultivation revealed that the spaces between the fibers—the pores of the scaffolds—were filled with cells (Figure 7b). Only very small parts, mostly the top of the fibers, were still visible.

**Figure 8:**
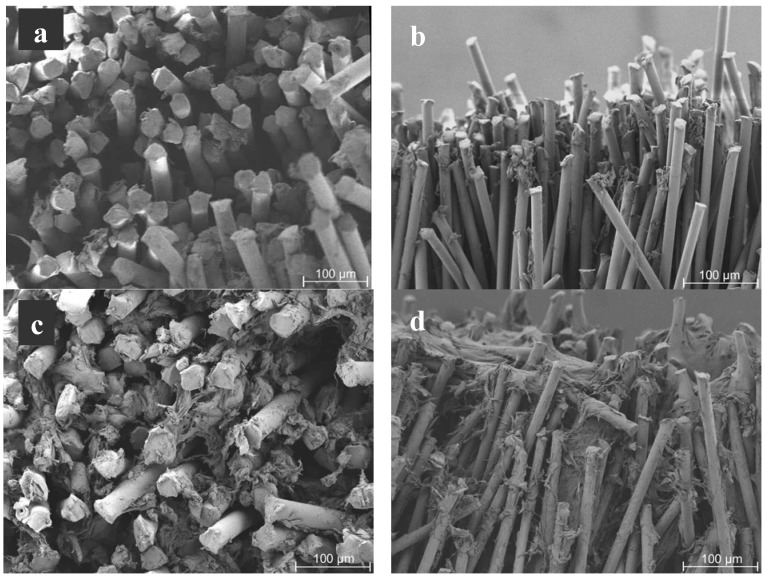
Scanning electron microscopy images of cell seeded samples with 1 mm fiber length (flocking time: 15 sec) at different time points of culture showing that flock scaffolds support proliferation of human mesenchymal stem cells. (**a**) Top view 7 days after seeding; (**b**) Longitudinal view day 7; (**c**) Top view 21 days after seeding; (**d**) Longitudinal view day 21. Magnification: Top views 200×; longitudinal views 100×.

Figure 8 shows images from different time points in culture. It can be seen that the amount of cells in the scaffolds increased. After 7 days of culture most parts of the fibers were still visible. Sporadic cells could be found along and between the fibers (Figure 8a,b). During culture, more and more of the fibers got covered by cells. 21 days after seeding (Figure 8c,d), significantly more cells were visible in the scaffolds than at the earlier time point.

The architecture of the scaffold with its high porosity and the huge pores described above as well as the nearly parallel arrangement of the fibers allow for an easy cell infiltration and a homogenous distribution of cells in the structures. They stretch between the fibers and fill up the pores. Differences in cell distribution could be observed between the scaffold types. In case of the flock scaffolds with the densest structure (1 mm fiber length, 15 s flocking time) most of the cells were found on top of the fibers directly after seeding. From there they migrated into the scaffold and finally distributed very homogeneously, filling the scaffold from top (fiber top) to bottom (adhesive layer). Because of the bigger pores in case of the other three investigated scaffold types the cells reached the adhesive layer already during seeding. So they filled the scaffold from the adhesive layer (bottom) to the fiber tops and also reached a uniform cell distribution over the whole scaffold with time.

**Figure 9:**
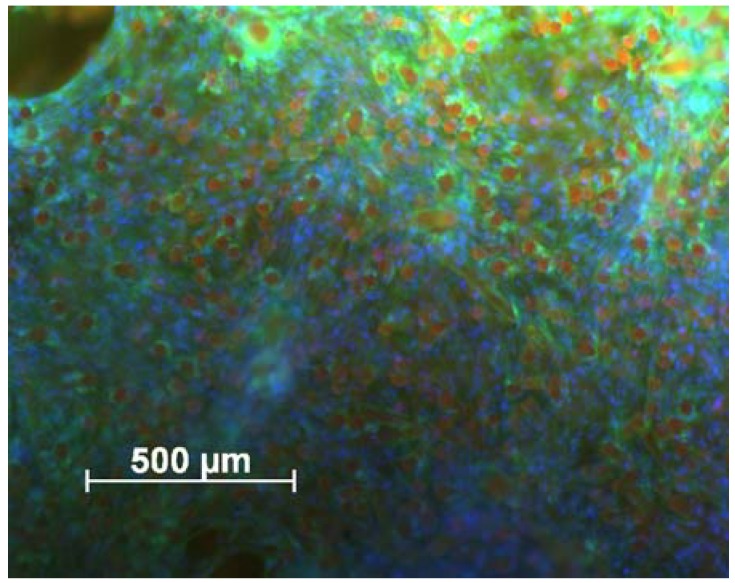
cLSM image of the scaffold surface 28 days after cell seeding. Cells were stained with DAPI/phalloidine. Actin fibers of cells appear green and nuclei of cells blue. Fibers of flock scaffold show up red because of their autofluorescence. The cells are distributed homogeneously over the surface. Scale bar represents 500 µm.

In addition to the investigation by SEM some samples were stained and analysed via fluorescence microscopy. Figure 9 shows an overview from the top of a cell seeded sample after 28 days of cell culture. Only the tips of the fibers were visible (red spots) due to autofluorescence and the spaces between the fibers were filled completely with cells.

This picture indicates that the cells are distributed very homogeneously over the whole scaffold surface. The microscopic evaluation revealed the suitability of flock scaffolds for tissue engineering. The scaffolds supported the adhesion of cells as well as their proliferation over time.

### 2.5. Cell Proliferation and Osteogenic Differentiation

**Figure 10:**
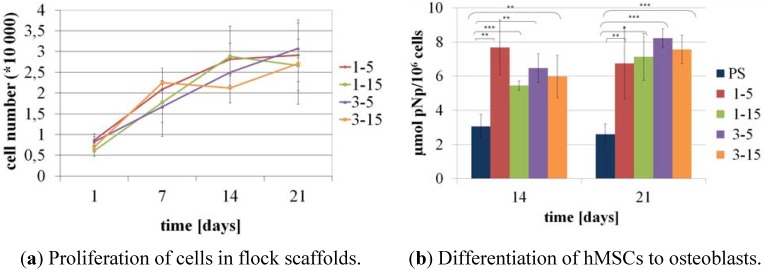
Proliferation and differentiation of hMSC in flock scaffolds with different fiber lengths and fiber distances. (**a**) Proliferation of osteogenically induced hMSC shown with the DNA-content of the scaffolds at time points 1, 7, 14, 21 days after seeding; (**b**) Specific ALP-activity of osteogenically induced hMSC on day 14 and 21 of cultivation. Graphs show mean (n = 3 bioconstructs) and standard deviation of the mean. *** *p* < 0.001; ** *p* < 0.01; * *p* < 0.05.

Proliferation of the cells in the flock scaffolds was investigated by quantification of the DNA content on days 1, 7, 14, and 21 of culture after lysis of the cell-seeded samples. DNA content was correlated with the cell number and showed an increase from day 1 to 21 of cultivation in all four scaffold types. After 21 days of cultivation the amount of differentiated cells has risen 5–6 fold compared to the initial amount of cells (Figure 10a).

The specific ALP activity—ALP activity measured from the scaffold related to the cell number determined from DNA content—of osteogenically induced cells in the flock scaffolds on day 14 and 21 of cultivation is shown in Figure 10b. In case of all four different scaffold types the specific ALP activity was significantly higher in the three-dimensional matrix of flock scaffolds compared to the control which was a normal polystyrene well plate. Between the four flock scaffold types no significant difference in specific ALP activity could be observed so that all investigated types of scaffolds support viability as well as osteogenic differentiation of hMSC.

Since no significant differences in proliferation and differentiation of the investigated structures were found and flock scaffolds produced with 1 mm fiber length and 15 sec flocking time showed the best results concerning mechanical properties (Figure 6) this scaffold type appears to be most capable for use in load-bearing tissues like bone. The nearly parallel arrangement of fibers and their function as compression members should protect cells cultivated in the scaffolds from critical mechanical loads.

In another study we were able, with the help of some co-workers, to show that flock scaffolds also support the chondrogenic phenotype [11]. Flock scaffolds were seeded with articular chondrocytes from porcine knees as well as porcine nucleus pulposus cells and it was demonstrated that these primary chondrocytes deposited a proteoglycan and collagen type II-rich ECM in flock scaffolds which means that cells kept their chondrogenic phenotype during cultivation.

In further experiments flock scaffolds were loaded with human MSC embedded in a collagen type I gel. It could be shown that chondrogenesis of MSC—determined by proteoglycan synthesis and collagen type II deposition of cells—loaded on flock scaffolds was significantly higher than that of MSC, cultivated only in collagen gels.

During compression testing and cell culture experiments no flock fibers detached themselves from the scaffold. This shows the stability of the novel scaffolds which is of major importance for tissue engineering applications and transplantation of constructs in the human body.

These encouraging results show that flock scaffolds represent a promising new matrix for tissue engineering, especially for load-bearing tissues like bone and cartilage. By changing the materials used for scaffold fabrication it should also be possible to create scaffolds for other tissues like skin—as, for example, the mineralized substrate we used in this study—could be replaced by a non-mineralized one.

## 3. Experimental Section

### 3.1. Fabrication of Flock Scaffolds

The scaffolds were fabricated as described elsewhere in detail [7]. To summarize briefly: A membrane made from mineralized collagen [12,13,14,15] was used as substrate. Before flocking the membranes were moistened with water and then covered with 20 wt-% gelatine dissolved in an aqueous 0.5 M NaCl solution as adhesive layer. To achieve a plane surface the overlaying gelatine was removed from the template with a coating knife. For flocking polyamide fibers (Swiss Flock, Stuttgart, Germany) with a length of 1 and 3 mm were used.

**Table 3:** Properties of polyamide fibers.

Length [mm]	Linear density [dtex]	Diameter [µm]	Slenderness ratio	Profile
1	6.7	30	6.7	round
3	22	50	7.3	round

These fibers were applied to the adhesive in an electrostatic field via flocking. For the flocking process a Maag RF 400/500 flocking machine (Maag Flockmaschinen, Gomaringen, Germany) was used with an acceleration voltage of 60 kV. The flocking time varied between 5 and 15 seconds. The flocking distance was 12 cm.

To stabilize the flock scaffolds they were crosslinked with *N*-(3-dimethylaminopropyl)-*N*’-ethylcarbodiimide (EDC) followed by freeze drying. For the present study scaffolds with a diameter of 5 or 11 mm were used. Scaffolds for cell culture experiments were sterilized by γ-irradiation before use.

### 3.2. Scanning Electron Microscopy (SEM)

For SEM the samples were freeze-dried and cut using a razor blade. The samples were mounted on specimen holders. Because of their poor conductivity, the samples were coated with carbon several times using a coating unit MED 010 (Bal-Tec, Balzers, Liechtenstein). SEM was carried out using a DSM 982 Gemini (Zeiss, Oberkochen, Germany) as well as an ESEM-FEG XL30 (Philips, Eindhoven, the Netherlands) operating in SEM mode, both at an acceleration voltage of 3 kV and a working distance of 5–10 mm detecting secondary electrodes.

### 3.3. Flock Density

For determining the flock density via SEM investigations, freeze dried samples were infiltrated with pure epoxy resin (according to [16]) by applying vacuum at room temperature. After polymerization at 60 °C for 72 h, samples were milled to appropriate shape (Leica EM Trim, equipped with diamond mill; Leica, Wetzlar, Germany). A flat surface of the block face of the sample was prepared on a Leica EM UCT ultra microtome (equipped with a Diatome diamond knife). Samples were mounted on SEM sample holders and carbon coated. Finally, the samples were analysed with a Philips ESEM-FEG XL 30 with backscatter electron detector. The acceleration voltage was 15 kV and working distance 5–12 mm. Flock density was determined by counting fibers per area.

### 3.4. Mechanical Testing

For compression testing scaffolds with a diameter of 20 mm were fabricated. Prior to measurement the scaffolds were incubated in water for 30 min to measure the samples in wet state. For compression testing, an electromechanical Instron 5,566 testing machine (static load cell 100 N) was used. The samples were compressed with a traverse speed of 4 mm/min in the case of 3 mm long fibers and 2 mm/min for 1 mm long fibers.

### 3.5. Cell Culture Experiments

Human mesenchymal stem cells (hMSC) were kindly provided by M. Bornhäuser and co-workers, Medical Clinic I, Dresden University Hospital.

The cells were expanded in Dulbecco’s Modified Eagle’s Medium (DMEM) low glucose containing 10% fetal calf serum, 10 U/ml penicillin, and 100 µg/ml streptomycin (all from Biochrom, Berlin, Germany) under standard conditions at 37 °C with 7% CO_2_.

Prior to seeding flock scaffolds were preincubated with cell culture medium for 24 hours. 6 × 10^4^ cells (for scaffolds with 11 mm diameter) or 3 × 10^4^ cells (for scaffolds with 5 mm diameter) were loaded on each scaffold. Medium was renewed every 3–4 days.

For osteogenic differentiation half of the samples were cultivated in differentiation medium based on DMEM supplemented with 10^−7^ M dexamethasone, 3.5 mM β-glycerophosphate and 0.05 mM ascorbic acid 2-phosphate (all from Sigma-Aldrich, Taufkirchen, Germany), 10 U/mL penicillin, and 100 µg/mL streptomycin (Biochrom). The cultivation was carried out at 37 °C in a humidified, 7% CO_2_ containing atmosphere.

After 1, 7, 14, 21 and 28 days of cultivation, samples were frozen for subsequent biochemical analysis (n = 3). Furthermore, samples were fixed with 3.7% formaldehyde in PBS for microscopic investigations.

### 3.6. Microscopic Evaluation of Cell Seeded Samples

#### 3.6.1. Scanning Electron Microscopy

Samples were fixed in 3.7% formaldehyde in PBS. After washing in PBS samples were dehydrated using a gradation series of ethanol/distilled water mixtures. For critical point drying a CPD 030 apparatus (Bal-Tec, Balzers, Liechtenstein) was used. The samples were then coated with carbon and gold (Bal-Tec MED 010) and investigated using a Philips XL 30 ESEM-FEG, operated in SEM mode.

#### 3.6.2. Confocal Laser Scanning Microscopy (cLSM) and DAPI/Phalloidine Staining

Cell-seeded and formaldehyde-fixed samples were permeabilized using 0.2% Triton X-100 (Sigma-Aldrich, Taufkirchen, Germany) in PBS and then rinsed five times in PBS. The autofluorescence of the bioconstructs was blocked by adding a 1% solution of bovine serum albumin (Sigma-Aldrich) in PBS. DAPI (1:1000, Sigma-Aldrich, St. Louis, MO, USA) stained the nuclei of the cells and Alexa Fluor 488 Phalloidine (1:40, Molecular Probes, Eugene, OR, USA) was used for staining the actin cytoskeleton. The samples were rinsed three times in PBS and then stored lightproof in distilled water at a temperature of 4 °C until microscopic investigation. Microscopy was performed using a cLSM 510 meta mounted on an upright Axioscope 2 FS mot (both Zeiss, Jena, Germany) and equipped with an additional NIR femtosecond-2-photons excitation unit (coherent Mira 900 F). DAPI was excited with NIR-fs-laser at 770 nm; Alexa Fluor 488 Phalloidine was excited with an argon ion laser at 488 nm.

### 3.7. Analyses of Alkaline Phosphatase (ALP) Activity and DNA Content

Cells were lysed with PBS containing 1% Triton X-100 for 50 min on ice. One aliquot of the lysed cell suspension was mixed with the ALP substrate solution, containing 1 mg/ml p-nitrophenyl phosphate, 0.1 M diethanolamine, 1% Triton X-100 (pH 9.8), and incubated at 37 °C for 30 min. To stop the enzymatic reaction 1 M NaOH was added. After centrifugation at 16,000 g for 10 min, the supernatants were transferred to a microtiter plate and absorbance was measured spectrophotometrically with a SpectraFluorPlus (Tecan, Crailsheim, Germany) at 405 nm and referred to a standard curve made from p-nitrophenol (Sigma-Aldrich, St. Louis, MO, USA).

Another aliquot of the cell lysate was mixed with the PicoGreen dsDNA quantification reagent (Molecular Probes) which was diluted 800 times in TE buffer (10 mM TRIS and 1 mM EDTA) and incubated for 5 min at room temperature in the dark. The intensity of fluorescence was measured using a SpectraFluorPlus plate reader (Tecan). The excitation wavelength was 485 nm and the emission was detected at 535 nm. Using a calibration line the measured relative fluorescence units were correlated to the cell number.

### 3.8. Statistical Analysis

All results are shown as mean ± standard deviation (SD). Statistical analyses were performed using a two-tailed, unpaired Student’s t-test. The levels of significance are marked as follows: * for *p* < 0.05, ** for *p* < 0.01 and *** for *p* < 0.001.

## 4. Conclusions

The present study deals with scaffolds for tissue engineering applications that were produced by electrostatic flocking which is a well-known textile technology. These flock scaffolds possess anisotropic properties. They show compression strength along the fibers and are flexible (depending on the substrate used) across the fibers which allows for good handling and adaption to tissue defects. This novel type of scaffold therefore combines favorable qualities which make it applicable for different tissues in the body. Because of the high compression strength in fiber direction, it appears that flock scaffolds are advantageous for load-bearing tissues like bone and cartilage.

We demonstrated that scaffolds produced by flock technology offer an oriented and open pore structure with a high surface-to-volume ratio that allows for easy seeding and migration of cells into the constructs. Flock scaffolds supported attachment and proliferation of hMSCs as well as differentiation along the osteoblastic lineage.

For an application in cartilage tissue engineering, in particular, non-mineralized substrates would be more suitable than the mineralized ones we used for the present study. First experiments with a non-mineralized collagen-membrane made from bovine pericardium—Lyoplant^®^ (B. Braun, Melsungen, Germany)—as substrate, were very promising and led to stable scaffolds. The encouraging results from the present, and also from a previous, study concerning the utilization of flock scaffolds for cartilage replacement [11] suggest that this new type of matrix might be suitable for the engineering of different tissues—merely needing to adapt materials used for scaffold fabrication to the respective tissue.

Further improvement of the scaffolds must be carried out to make them fully degradable. Promising results from preliminary experiments using fibers made of chitosan encourage our belief that this goal may well be achieved in the near future.
